# Laparoscopic treatment of median arcuate ligament syndrome in a 16-year-old male

**DOI:** 10.1016/j.ijscr.2018.10.006

**Published:** 2018-10-10

**Authors:** Yoshinori Fujiwara, Masaharu Higashida, Hisako Kubota, Yusakua Watanabe, Michi Ueno, Mio Uraoka, Yuko Okamoto, Shumei Mineta, Toshimasa Okada, Atsushi Tsuruta, Hiroaki Kusunoki, Tomio Ueno

**Affiliations:** a Department of Digestive Surgery, Kawasaki Medical School, Japan; b Department of Internal Medicine, Kawasaki Medical School, Japan

**Keywords:** MAL, median arcuate ligament, MALS, median arcuate ligament syndrome, Median arcuate ligament syndrome, Laparoscopic approach

## Abstract

**Introduction:**

MALS(Median Arcuate Ligament Syndrome) is rare disease. We experienced the case of MALS and successfully treated by laparoscopic approach.

**Presentation of case:**

A 16-year-old male was admitted to our hospital with postprandial epigastric pain that had been present for 3 years. Abdominal three-dimensional computed tomographic angiography showed stenosis of the celiac trunk, and abdominal Doppler ultrasonography showed that the blood flow in the celiac trunk varied between inspiration and expiration. Hence, the patient was diagnosed with median arcuate ligament syndrome. Laparoscopic decompression of the celiac trunk was performed by division of the ligament and partial excision of the celiac plexus. Intraoperative Doppler ultrasonography showed markedly improved flow in the celiac artery. The patient was discharged from hospital on postoperative day 7, and has no recurrent symptoms at 12 months postoperatively.

**Discussion:**

This case was most youngest male MALS treated by laparoscopic approach.

**Conclusion:**

Laparoscopic division of the median arcuate ligament is a minimally invasive, safe, and effective method for decompression of the celiac artery.

## Highlights

• Median Arcuate ligament(MAL) syndrome is rare disease.
• MAL causes compression and stenosis of the celiac artery and lead symptoms.
• 3-D CT angiography and doppler echogram is helpful for diagnosis.
• Laparoscopic approach is effective and less invasive for patients.
• Intraoperative doppler echogram is useful for confirmation of normal celiac arterial blood flow.

## 1 Introduction

Median arcuate ligament syndrome (MALS) is a rare disease in which a fibrous diaphragmatic arcuate ligament causes compression and stenosis of the celiac trunk. Extrinsic compression of the celiac trunk can lead to abdominal pain postprandially or during expiration. MALS can reportedly be effectively treated via resection of the arcuate ligament [1,2]. The number of cases in which arcuate ligament resection is performed laparoscopically is increasing due to advances in laparoscopic techniques [[3], [4], [5], [6]]. Herein, we present a case of a 16-year-old male in whom MALS was successfully treated via the laparoscopic approach. This case report was approved by ethical committee of Kawasaki Medical School (Authorization No 3166). The patient and his parents have provided permission to publish these features of his case, and the identity of the patient has been protected. The work has been reported in line with the SCARE criteria [7].

## 2 Case report

**Fig. 1:**
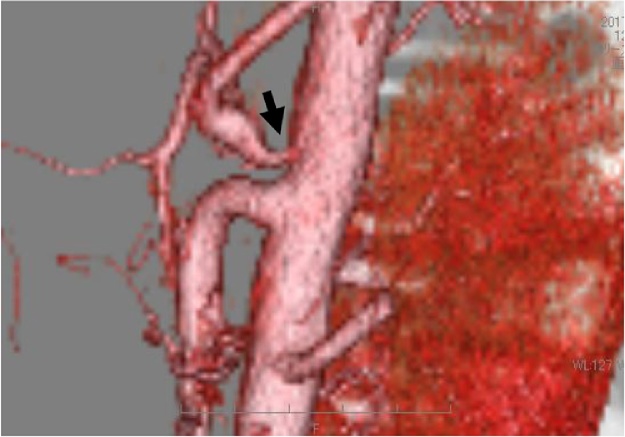
3D-CT angiography showed the stenosis of celiac artery(arrow).

**Fig. 2:**
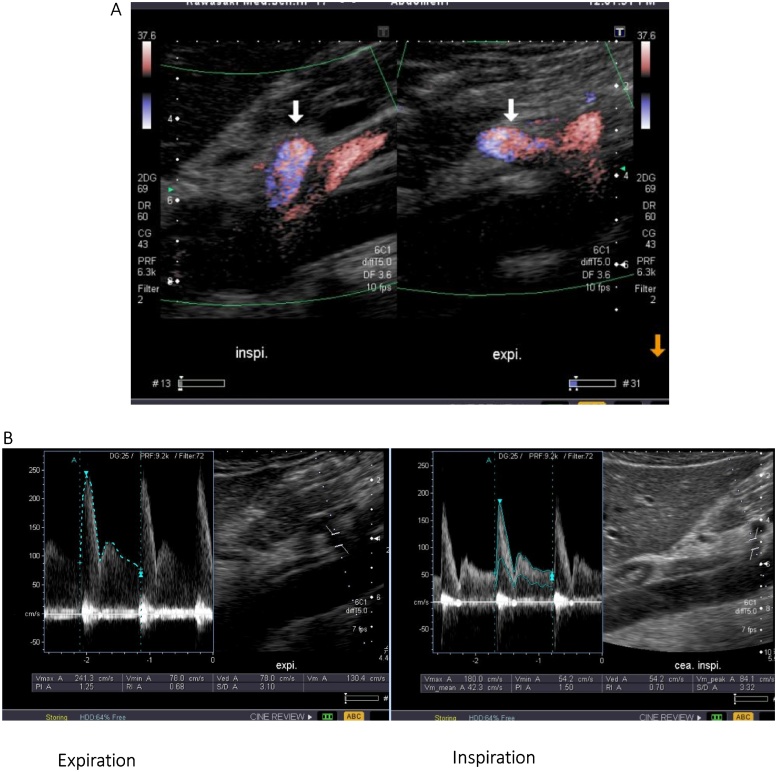
(A) Abdominal doppler echogram showed translocation and deformity of celiac artery between inspiration and expiration(arrow). (B) Abdominal doppler echogram showed the blood flow of celiac artery between inspiration and expiration.

A 16-year-old male presented at our hospital with postprandial abdominal pain that had been occurring for about 3 years. The patient was refusing to attend high school because of the abdominal pain, and he had symptoms of depression. Enhanced three-dimensional computed tomographic angiography of the abdomen showed stenosis of the celiac trunk (Fig. 1). An abdominal echogram showed translocation and deformity of the celiac artery between inspiration and expiration (Fig. 2A), and an abdominal Doppler ultrasonic echogram showed that the blood flow of the celiac trunk varied between inspiration and expiration (Fig. 2b). Hence, the patient was diagnosed with MALS. Informed consent was obtained from the patient and his parents, and laparoscopic ligament dissection was performed.

### 2.1 Operative procedure

**Fig. 3:**
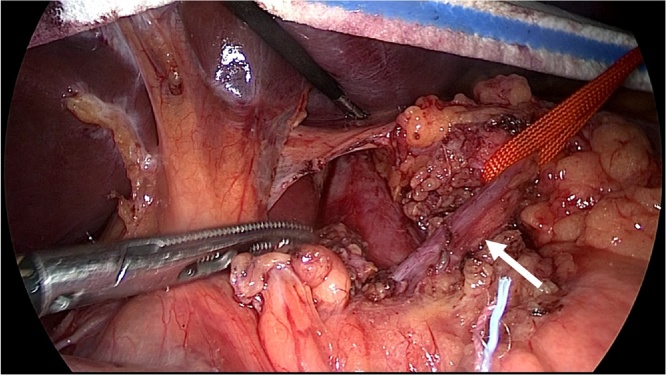
Left gastric artery and vein were identified and taping(arrow).

**Fig. 4:**
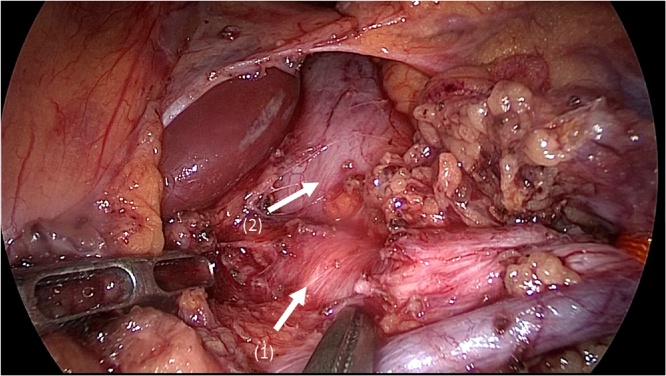
This picture showed the median arcuate ligament and nervous plexus around the celiac artery (arrow(1)) and thickened diaphragmatic crura(arrow(2)).

**Fig. 5:**
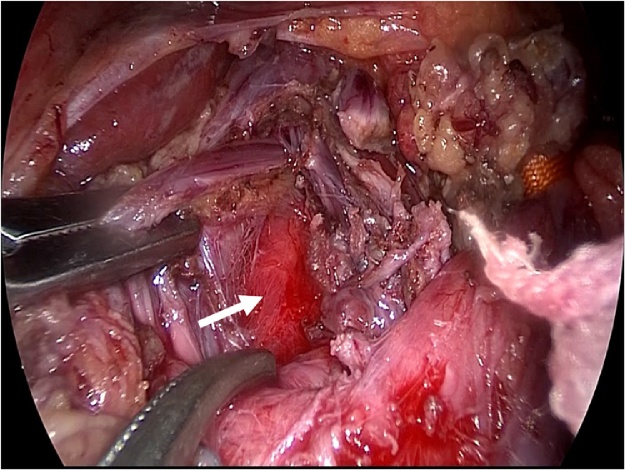
Diaphragmatic fibers were dissected and abdominal aorta was exposing(arrow).

**Fig. 6:**
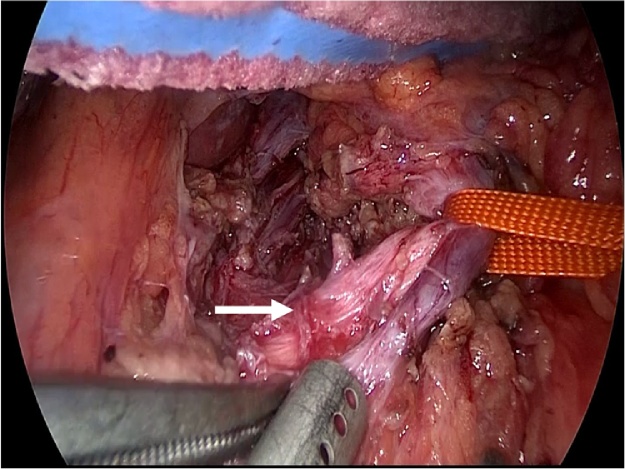
Median arcuate ligament and nervous plexus around the celiac artery were dissected(arrow).

**Fig. 7:**
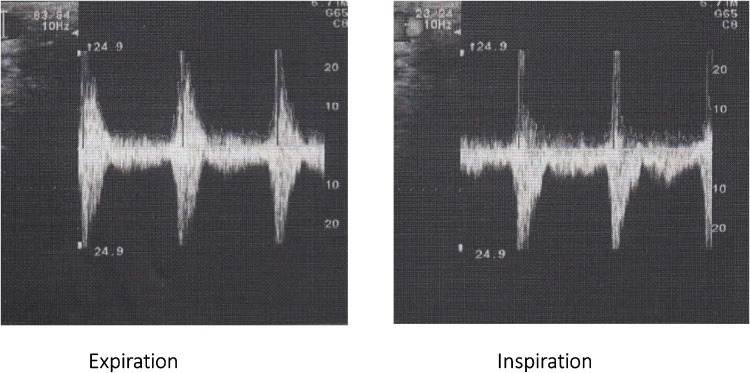
Intraoperative doppler ultrasonography showed the no difference of blood flow of celiac artery both inspiration and expiration.

**Fig. 8:**
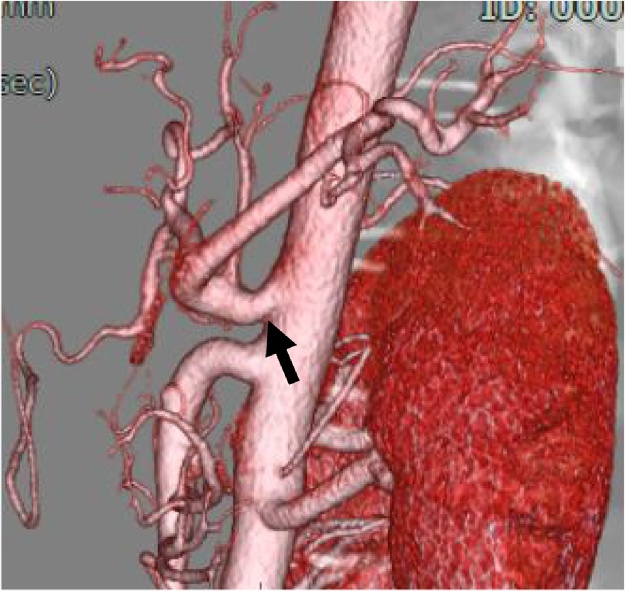
3D -CT angiography showed that celiac artery compression has been released(arrow).

General anesthesia was induced, and the patient was placed in the supine position with a 30° degree both legs opened. An open method was used to insert a 12-mm umbilical camera port, two 5-mm ports in the left upper abdomen, and a 10-mm and a 5-mm port in the right upper abdomen. Laparoscopy was performed with a 30° laparoscope (KARL STORZ Endoskope, Tokyo, Japan). After liver retraction, a Harmonic Scalpel^®^ (ETHICON, Tokyo, Japan) was used to open the minor omentum and dissect between the right diaphragmatic crus and the gastric ligament. The left gastric vein and artery were identified and taped (Fig. 3). The median arcuate ligament (MAL) and nervous plexus around the celiac artery were identified, and the thickened diaphragmatic crura were exposed (Fig. 4). The MAL and the nervous plexus were then dissected, and the dissection line was continued to the front of the abdominal aorta. The diaphragmatic fibers anterior to the aorta were dissected for approximately 5 cm in the cephalad direction, exposing about 4 cm of the abdominal aorta (Fig. 5, Fig. 6). Intraoperative Doppler ultrasonography confirmed that the celiac arterial blood flow no longer varied between expiration and inspiration (Fig. 7). After surgery, the patient’s symptoms were relieved, and decompression of the celiac artery was confirmed using three-dimensional computed tomographic angiography (Fig. 8). The patient was discharged from hospital on postoperative day 7, and has no recurrent symptoms at 12 months postoperatively.

## 3 Discussion

The MAL is a band of fibrous tissue that anteriorly connects the diaphragmatic crura surrounding the aortic hiatus. The diaphragmatic crura typically arise from the anterior aspect at the L1 to L4 vertebral level, and the celiac axis branches off the abdominal aorta at the vertebral level of Th11 to L1; however, wide variations in the origin of the celiac axis have been reported [8]. A higher origin of the celiac artery and/or a lower insertion of the diaphragm can result in compression of the celiac artery [9]. Asymptomatic MAL compression occurs in 10–25% of the general population. However, as only approximately 1% of these individuals with MAL compression develop MALS [9,10], few cases require treatment.

MALS is treated via decompression of the celiac artery. The mainstay of treatment involves an open surgical approach to divide or separate the MAL to relieve the compression of the celiac artery [1,2]. The diaphragmatic fibers should be incised for approximately 5 cm in a cephalad direction, exposing up to 4 cm of the aorta; confirmation of MAL release can then be done via visual inspection or using intraoperative Doppler ultrasonography. In the present case, we used ultrasonography to confirm that the celiac blood flow had almost the same velocity during expiration and inspiration.

Recent advances in laparoscopic techniques mean that the laparoscopic approach can be used to achieve celiac artery decompression [[3], [4], [5]]. Four surgical options are advocated for the treatment of MALS [11]: artery decompression and coeliac ganglionectomy, coeliac artery decompression and dilatation, coeliac artery decompression and reconstruction, and coeliac artery endovascular stenting. Rubinkiewicz et al. [12] evaluated the long-term results of 186 patients with MALS who were treated during 2002–2014, and concluded that the most frequently adopted MALS treatment is decompression of the celiac artery and coeliac ganglionectomy because of its positive long-term outcomes. We used the laparoscopic approach in the present case, and cut the diaphragmatic crus to expose approximately 4 cm of the abdominal aorta before performing partial coeliac ganglionectomy. Jimenez et al. [13] compared the laparoscopic and open approaches for MALS treatment performed between 1963 and 2012. They reported that the symptoms were relieved postoperatively in 85% of patients, and that the late recurrence rates were similar after laparoscopic versus open surgery (5.7% vs 6.8%) [13]; furthermore, postoperative complications only occurred in two patients in the laparoscopic group, compared with 6.5% of patients in the open group [13]. These findings suggest that the laparoscopic approach for MAL release might be as safe as open surgery, and provide a high rate of symptom relief. In addition, laparoscopic surgery reportedly results in shorter hospital stay and less intraoperative blood loss [14]. Due to recent advances in video image resolution, laparoscopic MAL release might become the main method used for MALS treatment.

MALS is a diagnosis of exclusion [9,15], and the most common symptoms are postprandial epigastric pain and weight loss. The present patient had experienced 3 years of postprandial epigastric pain and appetite loss, which prevented him from attending high school. He had also been diagnosed with depression by a neurologist, and was being medicated for this. After laparoscopic MAL release, the epigastric pain was resolved, and the patient was able to attend high school with no disease recurrence at 12 months postoperatively.

In summary, we reported the successful laparoscopic treatment of MALS in a 16-year-old male. The laparoscopic approach to divide the MAL and relieve celiac artery compression is recommended because of the shorter hospital stay, faster recovery, decreased blood loss, and better cosmetic outcomes compared with the open approach. Intraoperative Doppler ultrasonography is useful for identification of the celiac artery and confirmation of normalization of celiac arterial blood flow after the division of the MAL. Laparoscopic division of the MAL with intraoperative Doppler ultrasonography might become the standard treatment of MALS in the near future.

## Conflicts of interest

No conflicts of interest of all authors.

## Funding sources

No funding sources in all authors, and added in the text.

## Ethical approval

Ethical committee of Kawasaki Medical School was approved.

Authorization No 3166, which was added in the text.

## Consent

The patient and his parents have provided permission to publish these features of his case, and the identity of the patient has been protected at operation, and added in the text.

## Author contributions

Each authors approved this manuscript.

Data was prepared by Dr. Higashida and Kubota, and this operation was performed together.

Study design and manuscript was performed by Y. Fujiwara, and other authors read this manuscript and approved submission.

Dr. Ueno was decided final decision for submission, other authors discussed this manuscript together.

## Registration of research studies

This in not human study.

## Guarantor

The guarantor of this study was Dr. T. Ueno (Chief professor of our institution) and Y. Fujiwara (Professor of our institution).

## Provenance and peer review

Not commissioned, externally peer reviewed.

## References

[1] Dunbar J.D., Molnar W., Beman F.F., Marable S.A. (1965). Compression of the celiac trunk and abdominal angina. Am. J. Roentgenol. Radium Ther. Nucl. Med..

[2] Harjola P.T. (1963). A rare obstruction of the coeliac artery. Report of a case. Ann. Chir. Gynaecol. Fenn..

[3] Carbonell A.M., Kercher K.W., Heniford B.T., Matthews B.D. (2005). Laparoscopic management of median arcuate ligament syndrome. Surg. Endosc..

[4] Roayaie S., Jossart G., Gitlitz D., Lamparello P., Hollier L., Gagner M. (2000). Laparoscopic release of celiac artery compression syndrome facilitated by laparoscopic ultrasound scanning to confirm restoration of flow. J. Vasc. Surg..

[5] Vaziri K., Hungness E.S., Pearson E.G., Soper N.J. (2009). Laparoscopic treatment of celiac artery compression syndrome: case series and review of current treatment modalities. J. Gastrointest. Surg..

[6] Roseborough G.S. (2009). Laparoscopic management of celiac artery compression syndrome. J. Vasc. Surg..

[7] Agha R.A., Fowler A.J., Saeta A., Barai I., Rajmohan S., Orgill D.P., Afifi R., Al-Ahmadi R., Albrecht J., Alsawadi A., Aronson J., Ather M.H., Bashashati M., Basu S., Bradley P., Chalkoo M., Challacombe B., Cross T., Derbyshire L., Farooq N., Hoffman J., Kadioglu H., Kasivisvanathan V., Kirshtein B., Klappenbach R., Laskin D., Miguel D., Milburn J., Mousavi S.R., Muensterer O., Ngu J., Nixon I., Noureldin A., Perakath B., Raison N., Raveendran K., Sullivan T., Thoma A., Thorat M.A., Valmasoni M., Massarut S., D’cruz A., Vasudevan B., Giordano S., Roy G., Healy D., Machado-Aranda D., Carroll B., Rosin D. (2016). The SCARE statement: consensus-based surgical case report guidelines. Int. J. Surg..

[8] Loukas M., Pinyard J., Vaid S., Kinsella C., Tariq A., Tubbs R.S. (2007). Clinical anatomy of celiac artery compression syndrome: a review. Clin. Anat..

[9] Horton K.M., Talamini M.A., Fishman E.K. (2005). Median arcuate ligament syndrome: evaluation with CT angiography. RadioGraphics.

[10] Lindner H.H., Kemprud E. (1971). A clinicoanatomical study of the arcuate ligament of the diaphragm. Arch. Surg..

[11] Kohn G.P., Bitar R.S., Farber M.A., Marston W.A., Overby D.W., Farrell T.M. (2011). Treatment options and outcomes for celiac artery compression syndrome. Surg. Innov..

[12] Rubinkiewicz M., Ramakrishnan P.K., Henry B.M., Roy J., Budzynski A. (2015). Laparoscopic decompression as treatment for median arcuate ligament syndrome. Ann. R. Coll. Surg. Engl..

[13] Jimenez J.C., Harlander-Locke M., Dutson E.P. (2012). Open and laparoscopic treatment of median arcuate ligament syndrome. J. Vasc. Surg..

[14] De’Ath H.D., Wong S., Szentpali K., Somers S., Peck T., Wakefield C.H. (2018). The laparoscopic management of median arcuate ligament syndrome and its Long-term outcomes. J. Laparoendosc. Adv. Surg. Tech. A.

[15] Duncan A.A. (2008). Median arcuate ligament syndrome. Curr. Treat. Opt. Cardiovasc. Med..

